# In vivo stress reporters as early biomarkers of the cellular changes associated with progeria

**DOI:** 10.1111/jcmm.17574

**Published:** 2022-10-06

**Authors:** Francisco Inesta‐Vaquera, Florian Weiland, Colin J. Henderson, Charles Roland Wolf

**Affiliations:** ^1^ Division of Systems Medicine School of Medicine University of Dundee Jacqui Wood Cancer Centre Ninewells Hospital Dundee UK; ^2^ Department of Microbial and Molecular Systems (M^2^S) Centre for Food and Microbial Technology (CLMT) Laboratory of Enzyme Fermentation and Brewing Technology (EFBT) Technology Campus Ghent Ghent Belgium

**Keywords:** accelerated ageing, early biomarker, preclinical model, progeria, stress pathways

## Abstract

Age‐related diseases account for a high proportion of the total global burden of disease. Despite recent advances in understanding their molecular basis, there is a lack of suitable early biomarkers to test selected compounds and accelerate their translation to clinical trials. We have investigated the utility of in vivo stress reporter systems as surrogate early biomarkers of the degenerative disease progression. We hypothesized that cellular stress observed in models of human degenerative disease preceded overt cellular damage and at the same time will identify potential cytoprotective pathways. To test this hypothesis, we generated novel accelerated ageing (progeria) reporter mice by crossing the LmnaG609G mice into our oxidative stress/inflammation (Hmox1) and DNA damage (p21) stress reporter models. Histological analysis of reporter expression demonstrated a time‐dependent and tissue‐specific activation of the reporters in tissues directly associated with Progeria, including smooth muscle cells, the vasculature and gastrointestinal tract. Importantly, reporter expression was detected prior to any perceptible deleterious phenotype. Reporter expression can therefore be used as an early marker of progeria pathogenesis and to test therapeutic interventions. This work also demonstrates the potential to use stress reporter approaches to study and find new treatments for other degenerative diseases.

## 1 INTRODUCTION

The increase in morbidities associated with the increase in life expectancy is a major challenge to society. Such age‐related morbidities which include cardiovascular disease, cancer, degenerative and mental health disorders, account for 23% of the total global burden of disease.^1^ Investigation into the physiological causes of degenerative diseases and ageing have been extremely helpful in identifying therapies targeted at improving human health in old age.^2^ However, the biological complexity and temporal nature of the processes involved make the derivation of a reliable measure of ageing extremely difficult.^3^ Research in rare diseases of premature ageing (e.g. Hutchinson‐Gilford progeria syndrome, HGPS or Progeria) provides a powerful approach to understand the biological processes associated with ageing and the development of therapeutic interventions. However, the genetic complexity and phenotypic characteristics of these models often made this investigation high risk, expensive and does not allow multiple therapeutic interventions to be tested. There is therefore an urgent need for more informative early biomarkers for both mechanistic studies and to test therapeutic interventions.

Progeroid syndromes are a group of rare fatal genetic disorders that mimic clinical and molecular features of physiological ageing. In particular, Progeria is a fatal human disorder of accelerated ageing in children. Approximately 80% of cases carry a heterozygous silent point mutation—G608G—within exon 11 of the LMNA gene.^4^ Progeria symptoms begin within the first 2 years of life, and average life expectancy is 14.6 years.^5^ Patient characteristics are growth retardation, lipodystrophy, scleroderma, bone abnormalities (such as joint stiffening and impaired mobility due to osteodysplasia with osteolysis), alopecia and midface hypoplasia. Cardiovascular complications such as myocardial infarction or stroke due to premature atherosclerosis are the most frequent cause of death.^6^ Remarkably, there are several molecular features shared between accelerated and normal human ageing including accumulation of nuclear envelope defects, progerin accumulation and activation of inflammatory pathways as NF‐κB.^7^, ^8^

The LMNA G608G mutation creates an abnormal splice donor site, which produces a truncated protein (progerin) lacking residues 607–656 of prelamin A but retains the C‐terminal CAAX box, a target for prenylation. The result is an accumulation of a farnesylated aberrant progerin that triggers the many cellular changes and disease symptoms. These include nuclear defects as misshapen nuclear morphology, loss of peripheral heterochromatin, global changes in gene transcription and disrupted mitotic progression.^9^ LmnaG609G/G609G mice have marked similarities in the molecular and physiological alterations observed in Progeria patients: shortened life span, reduced body weight, bone and cardiovascular abnormalities, inflammation, metabolic deregulation and premature death at 14 weeks age.^5^ LmnaG609G/+ mouse models display only a weight loss phenotype after age of 35 weeks old, and they breed normally.

There have been significant advances using animal models in identifying potential therapeutic approaches to reduce the effects of progerin accumulation. These include the use of marketed drugs,^10^ diet interventions^11^ and new experimental in vivo gene editing.^12^, ^13^ Despite this, there is currently only one treatment approved for Progeria, lonafarniv, a farnesyl transferase inhibitor (FTI).^14^, ^15^, ^16^ lonafarniv, which has significant side effects, increased life expectancy by an average of 3 months through the first 3 years of treatment and by an average of 2.5 years through the maximum follow‐up time of 11 years.^14^ This treatment did not resolve lipodystrophy, skin features, alopecia and joint contractures, underscoring the fact that that FTI are not a cure of this disease.

One major barrier to progress in the treatment of this disease is the lack of robust models amenable for larger studies to identify and prioritize therapeutic approaches to be tested and then progressed to clinical trials. The development of early biomarkers prior to the onset of clinical symptoms would address this issue.

We have developed a portfolio of next‐generation mouse models in which the activation of cellular cytoprotective pathways, including inflammation/oxidative stress (NRF2‐Hmox1 reporter) and senescence/DNA damage (p53‐p21 reporter),^17^, ^18^ can be monitored at single‐cell resolution across all organs, tissues and cell types. Moreover, the measurements can also be performed non‐invasively and in real‐time. In each model, a short viral DNA sequence, known as a 2A sequence, is exploited to provide multiple reporters to be expressed (separately) from the endogenous gene promoter. For example, when Hmox1 reporter mice were exposed to oxidative stress inducers (e.g. paracetamol) or inflammatory mediators (e.g. LPS), a tissue‐ and cell‐specific induction of ß‐galactosidase reporter activity was detected. Similarly, when the p21 reporter mice were exposed to DNA damage‐inducing agents (e.g. gamma‐irradiation or cisplatin), an increase of p21 expression was detected at a high level of fidelity and resolution in target tissues. We hypothesized that the cellular stress resulting from accumulation of progerin, including DNA damage and inflammation, could activate the expression of our reporters.

In this paper, we report a time‐ and tissue‐specific increase in oxidative stress/inflammation and DNA damage reporter signals in disease‐relevant cell types before any deleterious phenotype is observed in LmnaG609G heterozygous and homozygous mice. We believe that these models provide a powerful approach to test potential therapies aimed at reducing the amount of accumulated progerin protein before the manifestation of clinical symptoms and without the need of complex measurements in mice during overall health decline.

## 2 METHODS

### 2.1 Animals

All animals used in this study were bred and maintained in the CIR Resource Unit, School of Life Sciences, University of Dundee. LmnaG609G animals were kindly provided by Prof. Carlos López‐Otín (Universidad de Oviedo, Spain), and they were described before.^9^ LmnaG609G_p21 and LmnaG609G_Hmox1 reporter mice were generated by crossing Lmna^G609G/+^ (Lmna^het^) mice into heterozygous Hmox1 (HOTT^het^) or p21 (p21^het^) reporter mice.^17^, ^18^ Mice were housed in open‐top cages in temperature‐controlled rooms at 21°C, with 45%–65% relative humidity and 12/12 h light/dark cycle. Mice had ad libitum access to food (R&M No.1 for stock females; R&M No. 3 for mating females; Special Diet Services) and water. Animals were regularly subjected to health and welfare monitoring as standard (twice daily). Environmental enrichment was provided for all animals. All animal work described was approved by the Welfare and Ethical use of Animals Committee of the University of Dundee. No regulated procedures were conducted in these animals.

### 2.2 Genotyping

Genotyping was performed as described before (ref.) with minor modifications. The primers used were as follows: Hmox1 reporter: HO1‐KI Fwd, 5_‐GCTGTATTACCTTTGGAGCAGG‐3_; HO‐1‐KI Rvr, 5′‐CCAAAGAGGTAGCTAATTCTATCAGG‐3′; p21 reporter: p21‐KI Fwd, 5′‐GCTACTTGTGCTGTTTGCACC‐3′; p21‐KI Rvr, 5′‐TCAAGGCTTTAGGTTCAAGTACC‐3′; LmnaG609G: Lmna1 5′‐AAGGGGCTGGGAGGACAGAG‐3′, Lmna3 5′‐AGCATGCAATAGGGTGGAAGGA‐3′.

### 2.3 Tissue harvesting and processing for cryo‐sectioning

Tissues were rapidly harvested postmortem and processed by immersion fixation in 4% para‐formaldehyde (PFA) (brain, small intestine, skin) for 2 h, 3% neutral‐buffered formalin (NBF) (liver) for 3 h or Mirsky's fixative (rest of tissues) for 24 h. For cryo‐sectioning, tissues were cryoprotected for 24 h in 30% (w/v) sucrose in phosphate‐buffered saline (PBS) at 4°C. Organs were embedded in Shandon M‐1 Embedding Matrix in a dry ice‐isopentane bath. Sectioning was performed on an OFT5000 cryostat (Bright Instrument Co.). With the exception of lung (14 μm) and brain (20 μm) sections, all sections were cut at 10 μm thickness.

### 2.4 In situ β‐galactosidase staining and histochemistry

Sections were rehydrated in PBS at room temperature for 15 min before being incubated overnight at 37°C in X‐gal staining solution: PBS (pH 7.4) containing 2 mM MgCl_2_, 0.01% (w/v) sodium deoxycholate, 0.02% (v/v) Igepal‐CA630, 5 mM potassium ferricyanide, 5 mM potassium ferrocyanide and 1 mg/ml 5‐bromo‐4‐chloro‐3‐indolyl β‐d‐galactopyranoside. On the following day, slides were washed in phosphate buffer solution, counterstained in Nuclear FastRed (Vector Laboratories) for 4 min, washed twice in distilled water for 2 min and dehydrated through 70% and 95% ethanol (4.5 and 1 min, respectively) before being incubated in Histoclear (VWR) for 3 min, air‐dried and mounted in DPX mountant (Sigma).

### 2.5 Western Blotting

Whole‐tissue protein extracts were prepared from snap frozen organs. Briefly, 300 μl of SDS‐lysis buffer (50 mM Tris–HCl pH 7.4, SDS 2%, EDTA 1 mM, EGTA 1 mM, β‐glycerophosphate 10 mM, pyrophosphate 5 m, sodium fluoride 50 mM, sodium‐ortho vanadate 1 mM and sucrose 0.27 M) supplemented with protease inhibitors and phosphatase inhibitors (Roche, 04693132001; SIGMA, P5726) were added per 100 mg of tissue and homogenized using a Polytron PT2100 benchtop homogenizer rotor. After 30 min, the resulting lysate was centrifuged for 15 min at 13,200 rpm on an Eppendorf tabletop centrifuge. Supernatant was recovered, and protein concentrations were measured by BCA protein assay kit (Bio‐Rad). Cell lysates were prepared in loading buffer followed by denaturalization. SDS‐PAGE and immunoblotting were carried out as previously described [38]. Antibodies used included Lamin A/C (Cell Signalling, 2032), Lemd2 (Sigma) and GAPDH (ThermoScientific). Licor Odissey scanner was used for data acquisition.

### 2.6 Proteomics analysis

Proteomic analysis was carried out by the Cambridge Proteomics Service. Tissue homogenates (SDS‐Lysis Buffer) were subjected to protein precipitation by overnight incubation in four times sample volume of cold ethanol and acetone at −20°C. Next day, samples were centrifuged at 16,000 g for 10 min (4°C) and supernatants discarded. Pellets were reduced, alkylated and trypsin digested before TMT Labelling (10‐Plex) according to manufacturer indications. Samples were then subjected to 1st dimension liquid chromatography, and 15 fractions were collected for LC–MS/MS (2 h gradients per run).

Mass spectrometry raw data were searched using MaxQuant (version 1.6.3.4) against a *Mus musculus* FASTA (canonical + protein isoforms, downloaded from Uniprot on 03/01/2021; 25,344 entries). Protease was set as Trypsin omiting the proline rule, variable modifications were set as oxidation of methionine, acetylation of the protein n‐terminus and farnesylation of cysteine, as fixed modification carbamidomethylation of cysteine was set. Output files were analysed by R (Version 4.1.1) using an in‐house developed script, adapted and modified from.^19^ In brief, within a replicate, the median intensity of all peptides belonging to a unique protein was taken to represent the respective protein abundance. These intensities were transformed and calibrated using variance stabilizing normalization (VSN). Statistical testing was carried out using limma^20^ under application of robust hyperparameter estimation. Additionally, the following R libraries were used: ggplot2, reshape2, vsn, limma,^20^ seqinr, plyr, stringr, ggrepel, ggpointdensity, wesanderson, scales and matrixStats. The version number of the used libraries is stored in the session info text file S3. Significance was assigned on a visual inspection of the derived volcano plots to avoid fixed pre‐determined (adjusted) *p*‐value cut‐offs.^19^ Mass spectrometry raw and search data have been deposited to PRIDE with the identifier PXD033518. Data analysis script files S1, S2 and session info files S3 are available from Zenodo (https://doi.org/10.5281/zenodo.6491287).

## 3 RESULTS

**FIGURE 1:**
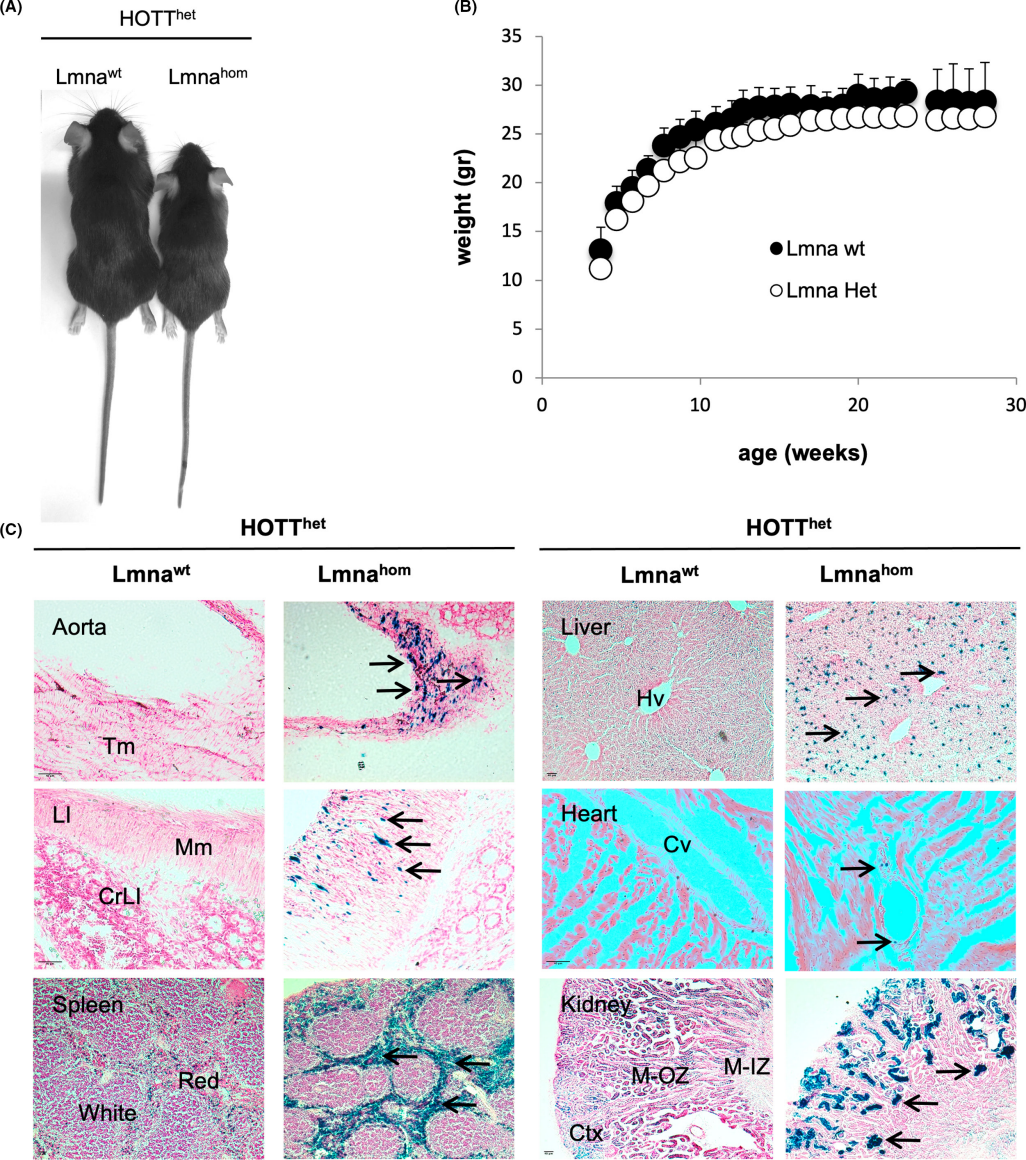
Demonstration of the HOTT reporter model to detect progerin expression. (A) Representative image of size differences found between Lmna^wt^‐HOTT^het^ and Lmna^hom^‐HOTT^het^ mice. (B) Female mice (HOTT^wt^) of indicated Lmna genotypes were weighted weekly for indicated times; (C) Tissues from triplicate HOTT^het^ reporters of indicated Lmna genotypes (10 weeks old) were harvested and in situ b‐galactosidase assay performed. Representative images for aorta (10x), large intestine (LI) (10x), spleen, kidney (2.5x) and heart (20x). Black arrows indicate ß‐galactosidase activity. Black scale bar: 40 μm. CrLI, crypts of Lieberkuhn; Cv, cardiac vessel; Hv, hepatic vessel; Mm, muscularis mucosa; Tm, tunica media. Red: red spleen; White: white spleen; Ctx: cortex; M‐OZ: medulla, outer zone; M‐IZ: medulla, inner zone

To establish whether the in vivo reporters of DNA damage or oxidative stress/inflammation can act as early biomarkers of degenerative disease progression, we generated LmnaG609G progeria—reporter mice.^9^, ^17^, ^21^ First, Lmna^het^ mice were crossed into HOTT^het^ as well as p21^het^ reporters resulting in the Lmna^het^‐HOTT^het^ or Lmna^het^‐p21^het^ lines. These were used as breeders for the duration of the project. During the course of our studies, we established a total of four no reporter Lmna^het^ × Lmna^het^ breeding pairs [18 litters, 81 pups; average size = 4.5 (±3.32) pups]; 11 breeding pairs of Lmna^het^‐p21^het^ × Lmna^het^‐p21^het^ mice [30 litters, 172 pups; average size = 5.3 ± 2.8 pups]; 13 breeding pairs of Lmna^het^‐HOTT^het^ × Lmna^het^‐HOTT^het^ [37 litters, 151 pups; average size = 3.9 (±2.8) pups]. The proportion of Lmna^het^ and Lmna^het^‐reporter^het^ mice generated was within the expected mendelian ratios. However, despite our intense breeding efforts, only four Lmna^hom^ (5%) and three Lmna^hom^‐HOTT^het^ (1.9%) mice were generated; moreover, no Lmna^hom^‐p21^het^ mice were born. However, the reasons for this are unclear, and there was no evidence of post‐natal mortality. Relative to HOTT^het^, Lmna^het^‐HOTT^het^ mice had a normal body size; however, Lmna^hom^‐HOTT^het^ mice were smaller and were euthanized before any health decline was observed (Figure 1A). As expected, Lmna^het^ mice did not display any early phenotype in the presence or absence of HOTT/p21 reporters (Figure 1B). Interestingly, for mice homozygous for the reporters only two Lmna^het^‐HOTT^hom^ (1.3%) and four Lmna^het^‐p21^hom^ (2.3%) mice were generated.

An initial study using Lmna^hom^‐HOTT^het^ reporter mice demonstrated a profound induction in disease‐relevant tissues, including aorta, large intestine (*muscularis* layer), spleen (white pulp), kidney (tubular cells), liver (Kupffer cells) and vasculature (e.g. small vessels in heart or kidney) (Figure 1C). Interestingly, despite the ubiquitous expression of Lamin A^22^ (for progerin expression, see Figure S1C), the reporter expression was predominantly observed in tissues with a large component of smooth muscle cells (aorta, large intestine, vasculature, see Figure S1A) or macrophages (white pulp spleen and Kupffer cells liver). The reporter expression pattern was consistent with previous reports indicating that smooth muscle cells are key cellular mediators in progeria development and targets for progeria treatment.^6^ The basal reporter expression and the increased signal due to progerin accumulation were consistent in male and female mice. Subsequently, animals of both sex were included in the reporter analysis of different age groups.

**FIGURE 2:**
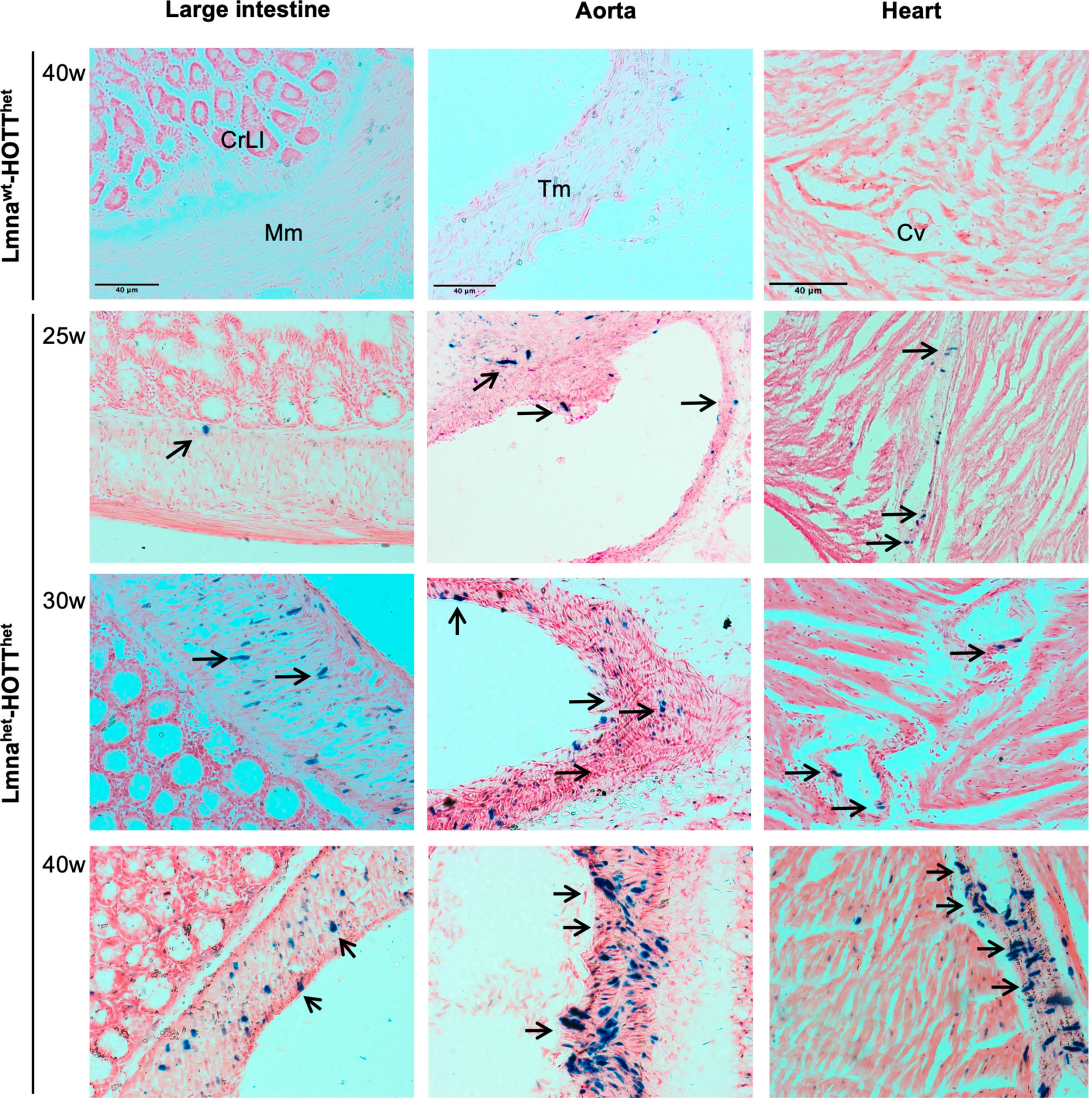
HOTT reporter activation in Lmna^het^ mice. Representative images of large intestine, aorta or heart tissues harvested from Lmna^wt^‐ or Lmna^het^‐HOTT^het^ reporter mice at indicated ages (*n* ≥ 3). w, weeks old; all images are 20x. Black arrows indicate ß‐galactosidase activity. Black scale bar: 40 μm. CrLI, crypts of Lieberkuhn; Cv, cardiac vessel; Mm, muscularis mucosa; Tm, tunica media

We next analysed the reporter expression of Lmna^het^‐HOTT^het^ mice in a more comprehensive panel of tissues and at different ages (16–40 wo). Strikingly, the presence of a single LmnaG609G allele was sufficient to induce the LacZ reporter in a time‐dependent and tissue‐specific manner (Figure 2). We observed marked reporter activation in smooth muscle cells in the large intestine and tissues vasculature, including aorta, heart or kidney (Figure 2 and Figure S1B). Expression of the reporter was first detected at 25 weeks of age, and the number of reporter‐positive cells increased in a time‐dependent manner up to 40 weeks of age. In contrast to mice homozygous null for the Lmna mutation (Figure 1C), reporter activation in Kupffer cells or spleen white pulp macrophages was not observed.

**FIGURE 3:**
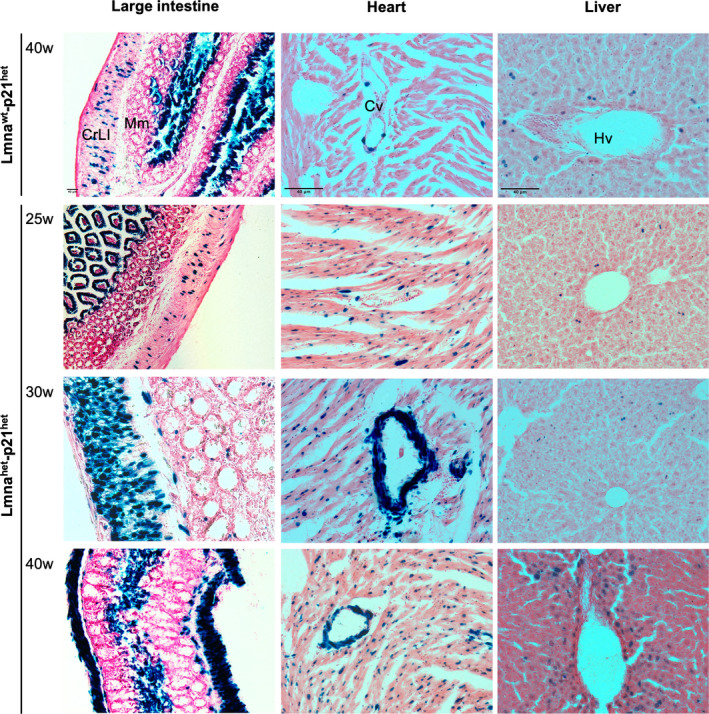
p21 reporter activation in Lmna^het^ mice. Representative images of large intestine, aorta or heart tissues harvested from Lmna^wt^‐ or Lmna^het^‐p21^het^ reporter mice at indicated ages (*n* ≥ 3). w, weeks old; all images are 20x, except large intestine (10x). Black arrows indicate ß‐galactosidase activity. Black scale bar: 40 μm. CrLI, crypts of Lieberkuhn; Cv, cardiac vessel; Hv, hepatic vessel; Mm, muscularis mucosa; Tm, tunica media

We then also analysed the expression of the DNA damage p21 reporter in a Lmna^het^‐p21^het^ mice at 25, 30 and 40 weeks old (Figure 3). As previously documented, the p21 reporter has a basal level of expression in a number of tissues including large intestine, heart and liver.^18^ At 25 wo, reporter activity in Lmna^het^‐p21^het^ mice was comparable to controls; however, an increase in reporter activity in smooth muscle cells (large intestine) and vasculature (e.g. heart vasculature) was observed at 30 wo and 40 wo. Interestingly, no further reporter activation was observed in liver, spleen or aorta (not shown) where increases of p21 expression have been reported in Lmna^hom^ mice.^9^ The p21 reporter therefore can detect effects of progerin accumulation in specific tissues and cell types. Moreover, the basal expression of both the p21 and HOTT reporters did not change with age, supporting the concept of induction of these stress response pathways as early events in progerin‐induced pathologies.

**FIGURE 4:**
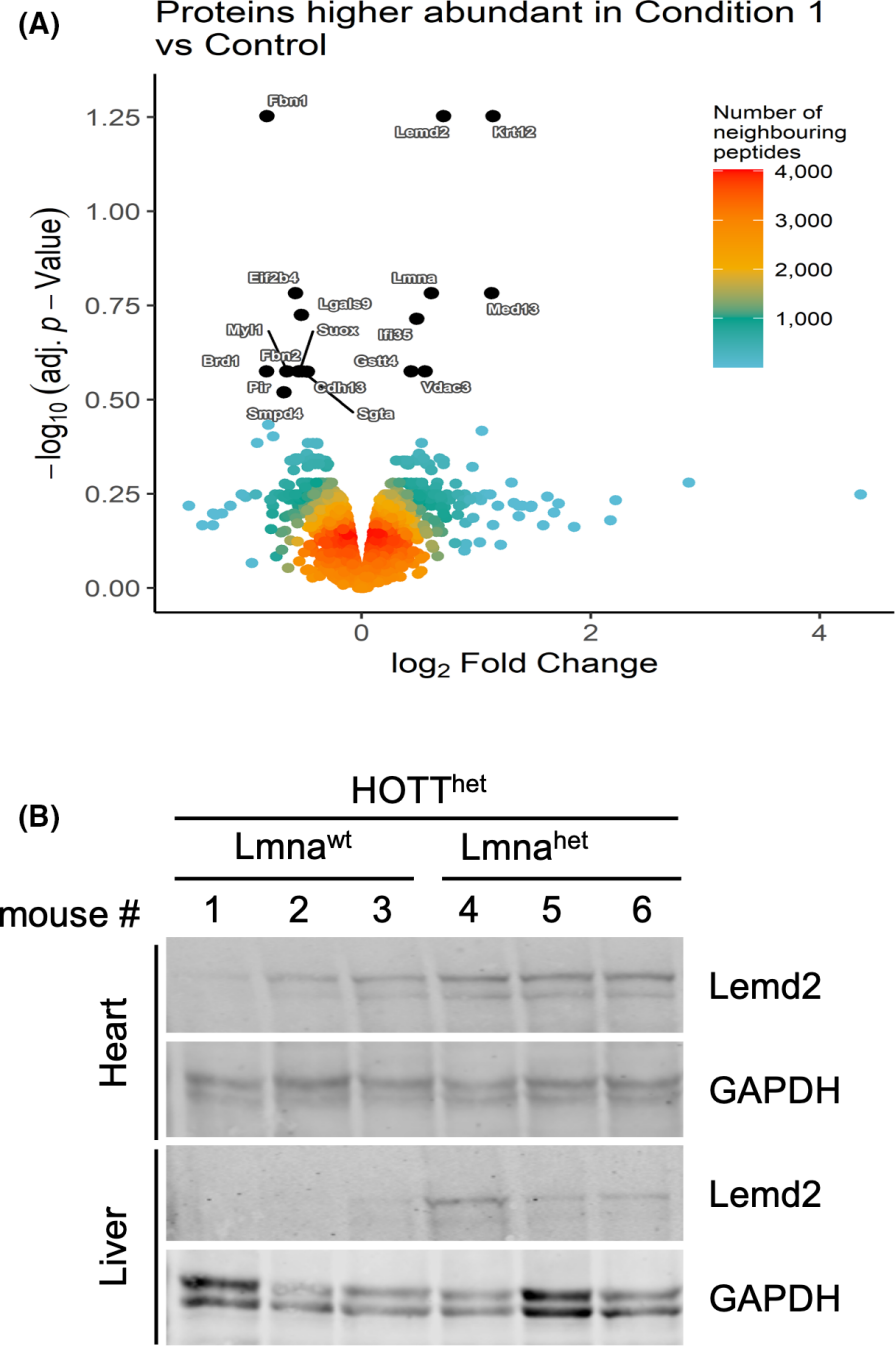
Identification of differently expressed proteins in Lmna^het^ mice. (A) Differential proteome analysis by TMT mass spectrometry. Volcano plot representing adjusted *p*‐values vs. fold change between Lmna^wt^‐HOTT^het^ vs. Lmna^het^‐HOTT^het^ heart tissues. Proteins of interest are labelled. Three biological replicates were analysed per genotype. (B) Immunoblots of indicated proteins from three independent whole tissue extracts per genotype

Finally, in order to investigate whether the transcriptional changes we observed using the reporter mice were also reflected in changes in protein expression, we carried out proteomic analysis on heart tissue obtained from Lmna^het^‐HOTT^het^ and were compared to Lmna^wt^‐HOTT^het^ mice (Figure 4). Triplicate Lmna^wt^‐HOTT^het^ and Lmna^het^‐HOTT^het^ tissues (25 wo mice) were solubilized and proteome analysis was carried out using TMT labelling. We identified 30,737 unique peptides from 4322 proteins (5% protein false discovery rate). Of those, there were three changes of particularly interest, including Lemd2, Krt12 (upregulation) and Fibrillin1 (downregulation). Interestingly, Lmnd2 (LEMD2), a previously described as a Lmna A associated protein, was induced in Lmna^het^ tissues.^23^ Conversely, the level of fibrillin‐1 a protein also related to cardiovascular diseases was reduced^24^ (Figure 4A). LEMD2 is an integral nuclear envelope inner membrane protein, implicated in the structural organization of the nuclear envelope and the localization of heterochromatin.^23^, ^25^ To validate these data, we measured the expression levels of Lemd2 protein in tissue homogenates by Western blot analysis. Lemd2 levels were increased in heart and liver of Lmna^het^‐HOTT^het^ mice compared to HOTT^het^ controls, confirming the proteomic findings (Figure 4B). Interestingly, a functional interaction between Lemd2 and Lmna A proteins has been described^23^, ^25^ and mutations in Lemd2 result in a nuclear envelopathy with early progeroid appearance.^26^, ^27^ It will be important to understand how the interaction of these proteins relates to the activation of stress pathways and progeria pathogenesis progression.

## 4 DISCUSSION

In this paper, we describe the application of stress reporter models as early markers of progeria pathogenesis. Importantly, reporter expression was detected prior to any perceptible deleterious phenotype, and it can therefore be used to test therapeutic interventions. This work also demonstrates the potential to use stress reporter approaches to study and find new treatments for other degenerative diseases associated with an old age.

The pattern of reporter's activation confirms previous studies that identified relevant tissues for progeria progression, which include those with a large component of smooth muscle cells such as the vasculature and large intestine.^6^ Indeed, a number of mechanisms have been proposed of how accumulation of progerin in smooth muscle cells results in loss of function and cell death. These include PARP1 downregulation, resulting in aberrant cell division (i.e. prolonged mitosis and caspase‐independent cell death).^28^ The loss of vascular smooth muscle cells can also induce further plaque vulnerability in atherosclerosis, which my lead to myocardial infarction and premature death,^6^ a characteristic of Progeria patients.

Our models demonstrate the molecular link between the NRF2 and p53 pathway with progerin accumulation. Interestingly, we obtained a lower‐than‐expected proportion of progerin‐reporter homozygous mice. We have previously reported a lower expression of the endogenous genes at the targeted gene locus (haplo‐insufficiency) which would significantly reduce their activity in homozygous reporter mice. Lower numbers of Lmna^hom^ mice generated based on Mendelian inheritance have been documented^29^; however, a deleterious interaction between these pathways and progerin accumulation cannot be ruled out. Indeed, HO‐1 plays a cytoprotective role in atherosclerosis progression by protecting vascular smooth muscle cells (a relevant cell type in progeria) against cell death induced by oxidative stress.^30^ Therefore, our breeding findings could reflect an enhanced susceptibility of Hmox1/p21 haplo‐insufficient mice to progerin accumulation and warrants further investigation. Interestingly, there is currently interest in evaluating the activation of the antioxidant NRF2 signalling pathway as a potential therapeutic approach in progeria.^31^

The mechanism of progerin‐mediated reporter activation remains unclear and will be the subject of future research. In the case of Hmox1, several pathways could be involved,^32^ including the transcription factor NRF2. Studies indicating a role of progerin in the modulation of the NRF2 pathway, together with its role in other cellular functions such as autophagy, have led to the proposal that pharmacological regulation of this pathway could be used as a therapy for progeria.^31^, ^33^, ^34^ Future genetic studies, for example, through the deletion of Nrf2 in the Lmna‐HOTT reporter mice would confirm its involvement. Hmox1 expression can also be induced by other signalling pathways activated by progerin.^32^ For instance, accumulation of progerin in the nuclear envelope has been observed in progeroid syndromes and is associated with an inflammatory response involving the activation of NF‐kB,^35^ and the development of several important features of progeroid phenotype. Importantly, defining the mechanistic links will help to prioritize the development of therapies.

Inflammation and senescence are phenotypes of Lmna^hom^ mice, reflected in the induction of associated markers such as p53 (p21) (senescence) and NF‐KB (e.g. IL‐6, CXCL1 and TNFa).^9^, ^35^ Our finding that the p53‐inducible gene p21 is induced in Lmna mice would support such effects. The fact that HOTT the reporter was activated several weeks earlier than the p21 reporter, suggests that oxidative stress/inflammation in smooth muscle cells is an earlier event and that p53 is activated as the phenotype progresses. It would be interesting to use pharmacological approaches to target these pathways at different stages of disease progression to dissect their importance on disease pathogenesis.

In addition to pathways leading to Hmox1 (e.g. NRF2 and NFKB) or p21 (e.g. p53) expression, other cellular stress responses have been associated with the accumulation of progerin. For example, transcriptional analysis of progerin expressing vascular smooth muscle cells revealed the differential expression of genes associated with to fibrosis, endoplasmic reticulum (ER) stress and the unfolded protein response.^33^ It should be noted, however, that in the majority of studies, these data have been obtained using a Lmna^hom^ genetic background. The toxicity of this severe phenotype could lead to simultaneous activation of multiple stress pathways (toxicity burst), making it difficult to define the molecular hierarchy of these events. We interrogated the proteomics changes associated with progerin in our models. Our analysis of Lmna^het^ mice cardiac tissue detected a nuclear protein, Lemd2, as significantly upregulated in progeria mice. Interestingly, Lemd2 has been shown to biochemically interact with Lmna A and Lemd2 mutations have been associated with the development of other laminopathies.^25^, ^26^ Further work will be required to establish the role of Lemd2 upregulation in progeria pathogenesis.

Our findings show a gene dosage effect on the severity of the progeria phenotype,^22^ which was also reflected in the activation of the reporters. The mechanism linking the reporter activation and the downstream deleterious toxicological effects of progerin accumulation are unclear and need to be further elucidated. They could be due to both direct (e.g. cellular stress in smooth muscle cells) or indirect effects such a production of inflammatory mediators by immune cells. Importantly, our studies demonstrate that transcriptional changes associated with stress signalling pathways are triggered by progerin accumulation prior to irreversible cellular damage. A causal connection between the presence of progerin and the loss of smooth muscle cells, especially in Lmna^hom^ mice has been reported.^28^ Therefore, the reporter activation provides an early biomarker of disease progression and provides a model system to develop therapies aimed at both reducing the amount of progerin and increasing the expression of cellular protective pathways or adjuvants in long‐term treatments. For example, it would allow intervention studies to evaluate the protective effect of high‐fat diet on reporter activity to be established.^11^

## 5 CONCLUSION

Stress response pathways reflecting oxidative stress/inflammation and DNA damage responses are activated in mouse models of progeria, with the induction of Hmox1 being an early event. The changes were observed in mice exhibiting none (Lmna heterozygous) or a mild (Lmna homozygous) progeria phenotype and occurred prior to any physiological changes. These reporter models provide a powerful approach to dissect out the biochemical changes involved in the pathogenesis of this disease and in the development of new treatments. They also have potential to understand the changes that occur in degenerative diseases and ageing in general.

## AUTHOR CONTRIBUTIONS

**Colin J. Henderson:** Project administration (equal); resources (equal); supervision (equal); writing – review and editing (equal). **Roland Wolf:** Conceptualization (equal); funding acquisition (equal); project administration (equal); resources (equal); supervision (equal); writing – review and editing (equal). **F. Inesta‐Vaquera:** Data curation (equal); formal analysis (equal); funding acquisition (equal); investigation (equal); methodology (equal); project administration (equal); resources (equal); validation (equal); writing – original draft (lead); writing – review and editing (lead). **Florian Weiland:** Data curation (equal); formal analysis (equal); investigation (equal); methodology (equal); software (equal); writing – review and editing (equal).

## CONFLICT OF INTERESTS

The authors declare no conflict of interest.

## Supporting information


Figure S1

## Data Availability

The data that support the findings of this study are available from the corresponding author upon reasonable request. Data analysis script files S1, S2 and session info files S3 are available from Zenodo;(https://doi.org/10.5281/zenodo.6491287).
