# Supplementary figures and images for: In vivo stress reporters as early biomarkers of the cellular changes associated with progeria
Source: J Cell Mol Med. 2022 Oct 6;26(21):5463–72. doi: 10.1111/jcmm.17574 (PMC9639039; doi:10.1111/jcmm.17574)

**A**

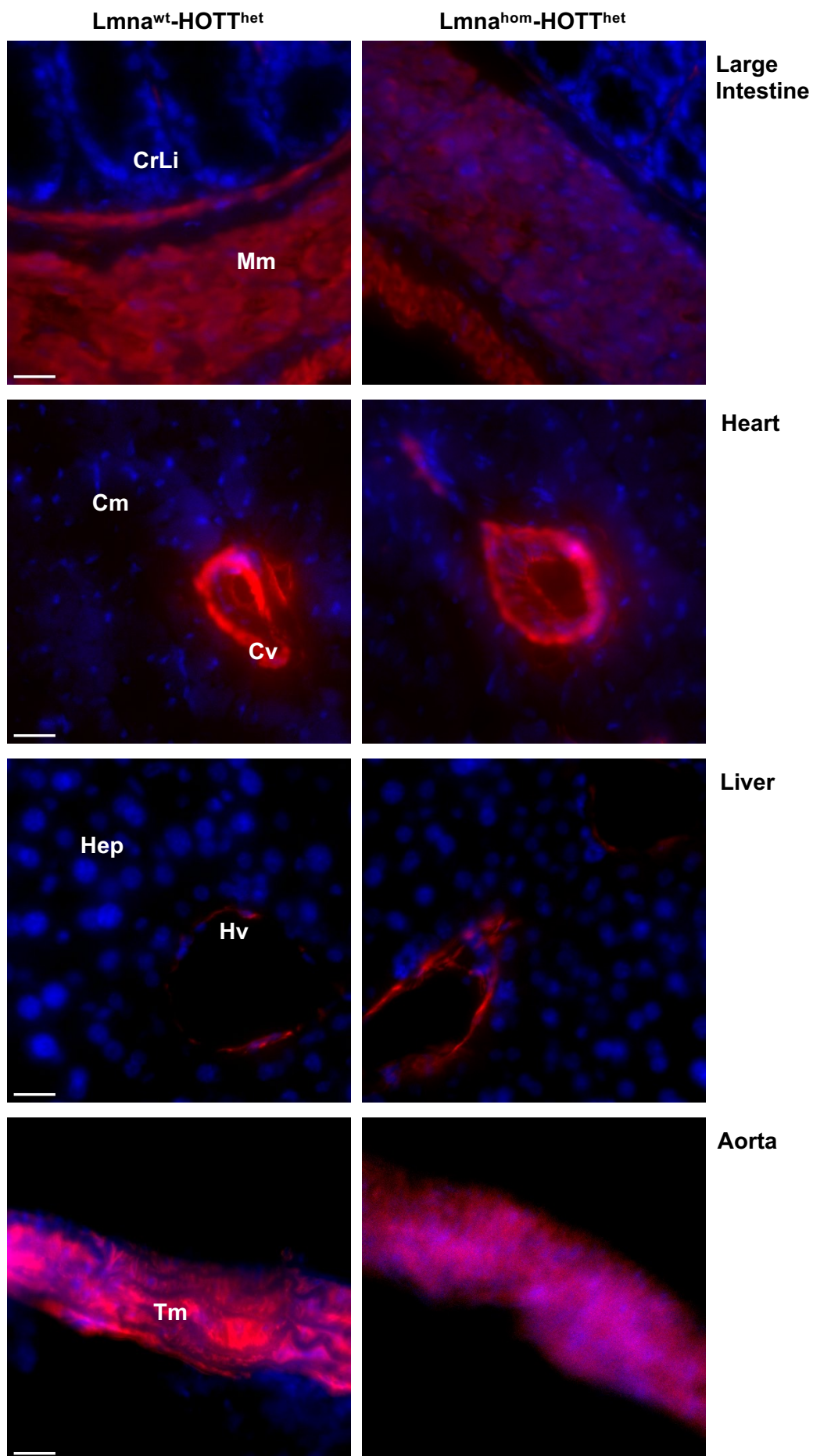

**Figure Supplementary 1.**

B

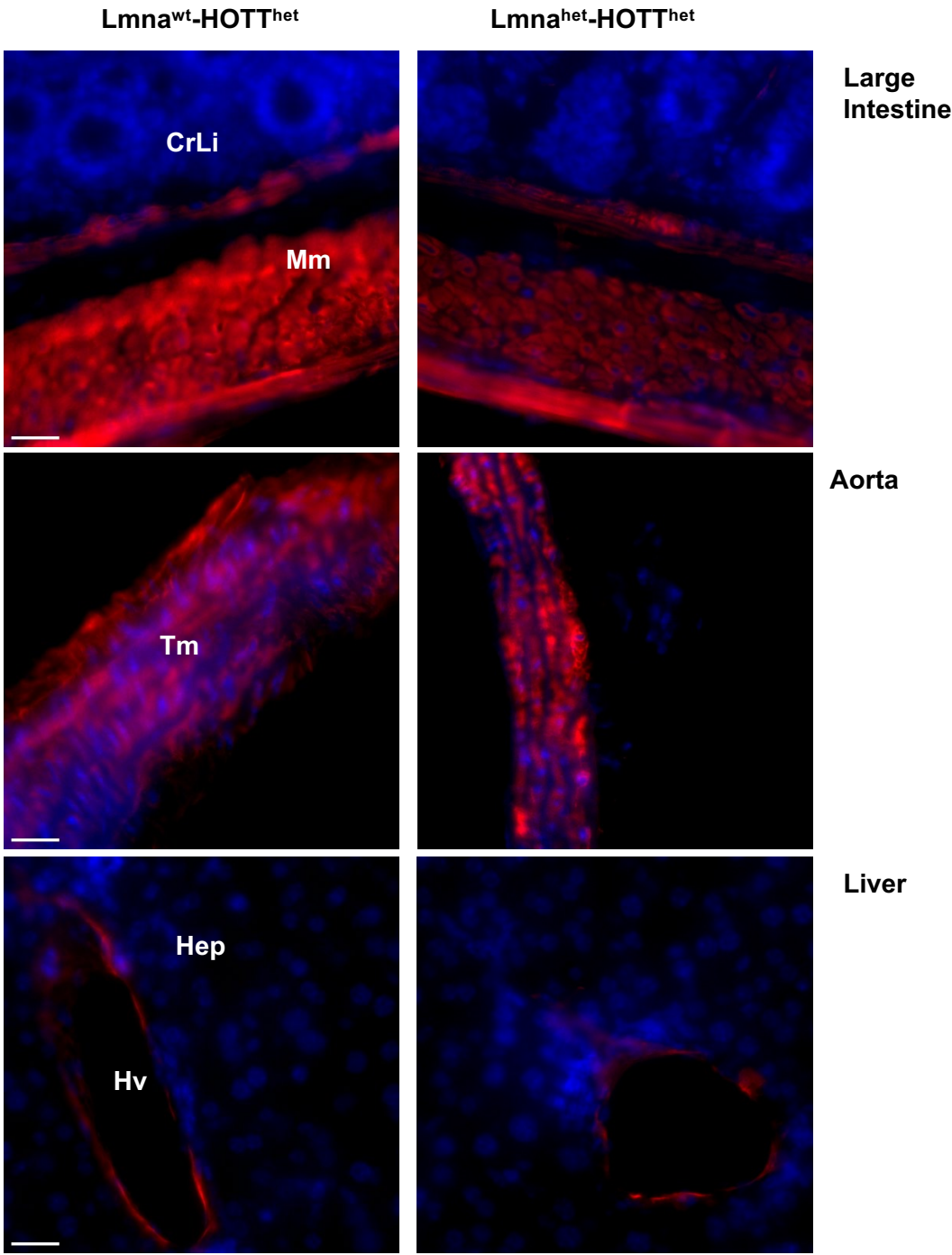

Figure Supplementary 1.

C

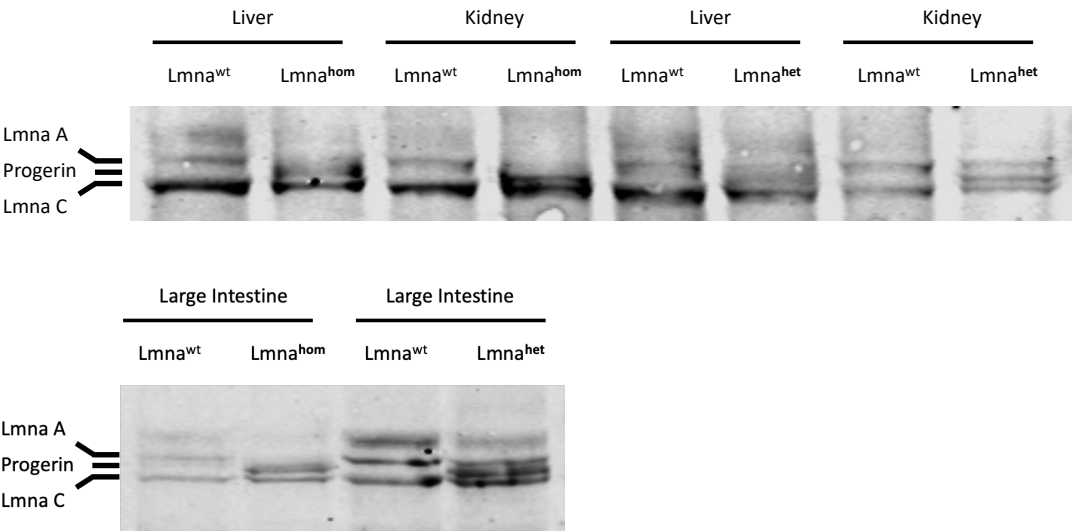

Figure Supplementary 1.

Supplement: Supplementary file 1 — Figure S1 [file JCMM-26-5463-s001.pdf]
